# An Account of Some Extraordinary Effects Arising from a Sting of a Wasp

**Published:** 1819-06

**Authors:** 


*For the London Medical and Physical Journal.*

### *An Account of some extraordinary Effects arising from a Sting of a Wasp;* by Chirurgus.

MR. A., aged 40, in the month of August last, was stung in the middle-finger of the right hand by a wasp, which deprived him of all sense and power of muscular action. He felt, at the instant, as if he had been pierced at every joint by a sharp instrument; or, more properly, it conveyed to his mind a sensation like the shock of electricity, though attended with much more acute pain. His hand, arm, face, and head, became very much swoln; the eyes were suffused with blood, so much so, that the tunicæ adnatæ were as red as in acute ophthalmia; and he complained of intense smarting and itching pain in them. The course of the absorbents of the arm could be traced by red lines; and he was also tormented by a very distressing itching all over his body. After the lapse of ten minutes from the receipt of the accident, I was called to him, and found him in a state of total insensibilty, except that he was occasionally roused by the action of the stomach to vomit; and then immediately relapsed into the same state, expressing a strong desire to lie down and sleep. The action of the heart was extremely feeble, and the pulse at the wrist scarcely perceptible. I thought it improper that he should be permitted to sleep; and taking an arm myself, and giving the other to an attendant, we walked, or rather dragged, him about, for three-quarters of an hour, when he gradually recovered. As soon as he could swallow, I gave him some of the volatile alkali. The hand and arm, however, remained swoln and painful; and he complained of much languor and uncomfortable feelings for many days.

I never met with such severe symptoms from the sting of a wasp; and, upon enquiry of many of my medical friends, they assure me of the same.

I must also mention, I was lately called to another person under nearly similar circumstances; but the symptoms were not quite so violent.

Mr. A. was stung about five years ago, when the like effects took place, though they were not then so severe.

*Twickenham; Oct.* 3, 1818.

